# Profound downregulation of neural transcription factor Npas4 and Nr4a family in fetal mice neurons infected with Zika virus

**DOI:** 10.1371/journal.pntd.0009425

**Published:** 2021-05-28

**Authors:** Sergio P. Alpuche-Lazcano, James Saliba, Vivian V. Costa, Gabriel H. Campolina-Silva, Fernanda M. Marim, Lucas S. Ribeiro, Volker Blank, Andrew J. Mouland, Mauro M. Teixeira, Anne Gatignol

**Affiliations:** 1 Virus-Cell Interactions Laboratory, Lady Davis Institute for Medical Research, Montréal, Canada; 2 RNA Trafficking Laboratory, Lady Davis Institute for Medical Research, Montréal, Canada; 3 Department of Medicine, Division of Experimental Medicine, McGill University, Montréal, Canada; 4 Lady Davis Institute for Medical Research, Montréal, Canada; 5 Departamento de Bioquimica e Imunologia do Instituto de Ciencias Biologicas, Universidade Federal de Minas Gerais, Belo Horizonte, Brazil; 6 Departamento de Morfologia do Instituto de Ciencias Biologicas, Universidade Federal de Minas Gerais, Belo Horizonte, Brazil; 7 Department of Medicine, Montréal, Canada; 8 Department of Physiology, McGill University, Montréal, Canada; 9 Department of Microbiology and Immunology, McGill University, Montréal, Canada; Universidade Federal de Minas Gerais, BRAZIL

## Abstract

Zika virus (ZIKV) infection of neurons leads to neurological complications and congenital malformations of the brain of neonates. To date, ZIKV mechanism of infection and pathogenesis is not entirely understood and different studies on gene regulation of ZIKV-infected cells have identified a dysregulation of inflammatory and stem cell maintenance pathways. MicroRNAs (miRNAs) are post-transcriptional regulators of cellular genes and they contribute to cell development in normal function and disease. Previous reports with integrative analyses of messenger RNAs (mRNAs) and miRNAs during ZIKV infection have not identified neurological pathway defects. We hypothesized that dysregulation of pathways involved in neurological functions will be identified by RNA profiling of ZIKV-infected fetal neurons. We therefore used microarrays to analyze gene expression levels following ZIKV infection of fetal murine neurons. We observed that the expression levels of transcription factors such as neural PAS domain protein 4 (Npas4) and of three members of the orphan nuclear receptor 4 (Nr4a) were severely decreased after viral infection. We confirmed that their downregulation was at both the mRNA level and at the protein level. The dysregulation of these transcription factors has been previously linked to aberrant neural functions and development. We next examined the miRNA expression profile in infected primary murine neurons by microarray and found that various miRNAs were dysregulated upon ZIKV infection. An integrative analysis of the differentially expressed miRNAs and mRNAs indicated that miR-7013-5p targets Nr4a3 gene. Using miRmimics, we corroborated that miR-7013-5p downregulates Nr4a3 mRNA and protein levels. Our data identify a profound dysregulation of neural transcription factors with an overexpression of miR-7013-5p that results in decreased Nr4a3 expression, likely a main contributor to ZIKV-induced neuronal dysfunction.

## Author summary

Zika virus (ZIKV) is an emerging virus transmitted horizontally between humans through mosquito bites, and sexual intercourse generally inducing a mild disease. ZIKV is also transmitted vertically from mother-to-child producing congenital ZIKV syndrome (CZVS) in neonates. CZVS leads to severe microcephaly associated with neurological, ocular, musculoskeletal, genitourinary disorders and other disabilities. Although numerous studies have been performed on ZIKV infection of brain cells, we are still far from understanding how ZIKV infection leads to dysregulation of host genes, virus-induced cytopathicity and consequent pathology. Micro (mi)RNAs are small noncoding RNAs encoded and processed by the host cell. They regulate gene expression at the post-transcriptional level in a process called RNA interference (RNAi). Here, we evaluated the relationship between ZIKV infection and the level of mRNAs and miRNAs expressed in the cell. ZIKV infection of mouse embryo neurons downregulated several neural immediate-early genes (IEG). Moreover, we revealed that ZIKV infection led to aberrant regulation of several miRNAs, and identified one whose cognate target was a neural IEG. Our work identifies novel genes and miRNAs that are modulated upon ZIKV infection of fetal murine neurons, therefore linking neuronal dysfunction to transcription and the RNA interference pathway.

## Introduction

Zika virus (ZIKV) is an emerging flavivirus transmitted mainly by the *Aedes aegypti* mosquito [1]. ZIKV was first isolated from a sentinel rhesus monkey in Ziika forest of Uganda in 1947 [2]. By 1966, it reached Asia and remained silent for decades until 2013 when it triggered a large outbreak in French Polynesia and shortly after reached Brazil, Latin America and the USA [3–6]. ZIKV infection induces mild symptoms in adults such as fever, headache, rash, conjunctivitis and arthralgia [7]. ZIKV is also associated with neurological complications including Guillain-Barré syndrome and congenital brain abnormalities such as microcephaly in neonates called congenital ZIKV syndrome (CZVS) [8–12]. Although the pandemic in mid-2021 has dramatically decreased, sporadic cases continue to occur in Brazil and could give rise to large outbreaks in the future. While antiviral strategies continue to be explored, to date neither a vaccine nor a suitable treatment is available [13–15].

ZIKV belongs to the Flaviviridae family with a single positive sense monocistronic RNA of 11kb, which is translated into a single polyprotein. The polyprotein contains structural domains (C, prM and E) and non-structural domains (NS1, NS2A, NS2B, NS3, NS4A, NS4B and NS5) that are cleaved by the viral NS2B-NS3 protease and cellular peptidases including furin [16–20]. Structural and non-structural proteins of ZIKV share functions and characteristics with other flaviviruses. However, the cell response can change in comparison to other members of the Flaviviridae family [21–25]. The E protein of ZIKV mediates its attachment to cellular receptors like Ax1, Tim-1, Tyro3 and DC-SIGN present on the surface of various host cell types, including epidermal, immune, retinal cells and neurons [26–35]. This binding triggers clathrin-mediated endocytosis of the virion. This step is followed by viral replication in the endoplasmic reticulum (ER), similar to that of other flaviviruses [7,36–39].

The interaction of ZIKV with cellular pathways such as the RNA interference (RNAi) pathway has not been completely explored. This pathway leads to the repression of messenger RNA (mRNA) expression through small RNA transcripts of 22 nucleotides called microRNA (miRNAs). In humans, target site analysis predicted that miRNAs silence more than 60% of protein-coding genes [40]. MiRNAs modulate mRNAs continuously regardless of the state of the cell, be it during development, disease states, tumorigenesis or during viral infections [41–46].

Few studies have described the mRNA and miRNA expression profiles in ZIKV-infected cells [47–49]. Furthermore, the relationship between cellular RNA pathways and ZIKV pathogenesis during CZVS has not been explored. To date, RNA sequencing analysis of ZIKV-infected astrocytes have resulted in an upregulation of miRNAs with antiviral properties and of genes involved in the unfolded protein response pathway in the ER [47]. In another study, ZIKV infection of primary murine neurons followed by mRNA and miRNA screening revealed a significant upregulation of genes involved in the inflammatory response [48]. Furthermore, Argonaute crosslinking and immunoprecipitation (Ago-CLIP) RNA followed by sequencing analysis in ZIKV-infected human neuroprogenitor cells (NPCs) revealed miRNA-mediated repression of genes involved in neurogenesis and stem cell maintenance [49].

In this study, we hypothesized that genes involved in neural functions could be dysregulated by the inappropriate expression of miRNAs during the infection of fetal brain by a recent ZIKV strain. To verify this hypothesis, we infected primary murine fetal neurons with the ZIKV strain HS-2015-BA-01 isolated in Brazil that was previously shown to be highly cytopathic [50,51]. Total RNA was extracted from the infected neurons to determine the mRNA and miRNA profiles using bioinformatics tools. We identified that neural PAS domain protein 4 (Npas4) and the orphan nuclear receptor 4 (Nr4a) transcription factors were profoundly modulated upon ZIKV infection. Remarkably, we found that Nr4a3 was downregulated by the overexpression of miR-7013-5p, establishing a link between the miRNA pathway and the regulation of mRNA expression of genes involved in neural development and function.

## Material and methods

### Ethics statements

C57BL/6 mice of 25-30g were housed at 23°C on a 12 hour light/12 hour dark cycle following the recommendations of the Brazilian Government (law 11794/2008a) and approved by the Committee on Animal Ethics of the UFMG (CEUA/UFMG, permit protocol no. 242/2016).

### Neuronal cell cultures and cell lines

Pregnant mice delivered 6–9 mouse embryos on day 15 which were dissected to prepare the cerebral cortex and striatal region. After dissection, the brain tissue was submitted to trypsin digestion followed by cell dissociation using a fire-polished Pasteur pipette. After dissociation, a pool of cells from the cortex and striatal region of all embryos of each mother was plated on previously polyL-ornithine-coated dishes with Neurobasal medium (Thermo Fisher Scientific) supplemented with N2 and B27, 2 mM GlutaMAX (Thermo Fisher Scientific), 50 μg/mL streptomycin, and 50 U/mL penicillin (Gibco), incubated at 37°C and 5% CO_2_ in a humidified incubator and cultured for five days, as previously described [52].

Mouse neuroblastoma cell line Neuro-2A (ATCC, CCL-131) was donated by Dr. Stéphane Richard (Lady Davis Institute, Montréal, Canada). Neuro-2A cells were maintained in Dulbecco’s modified Eagle’s medium (D-MEM) (Hyclone) supplemented with 10% fetal bovine serum (FBS) (Hyclone), 50 μg/mL streptomycin, and 50 U/mL penicillin (Gibco).

### ZIKV strain and virus infection

ZIKV strain HS-2015-BA-01 (Genbank accession KX520666.1) was isolated in August 2015 in Salvador, Bahia from the blood of a female patient with a cutaneous rash, muscular pain and low-grade fever. It was passaged three times in *Aedes albopictus* C6/36 mosquito cell lines (ATCC, CRL-1660), once in Vero E6 cells (ATCC, CRL-1586) and titrated by plaque assay as described [50,52,53]. Neurons derived from mouse embryos were infected for 1 hour with ZIKV at multiplicity of infection (MOI) of 1. After the adsorption time, the medium containing ZIKV was replaced by complete Neurobasal medium and after 6 or 24 hours, the culture was submitted to different analyses such as: a) cell viability assessed by LIVE/DEAD Cell Viability Assay; b) viral quantification by plaque assay; c) Western blot analyses; and, d) immunofluorescence. Mock was used as a negative control group in which cells received only medium. In some experiments, inactivated ZIKV (30 minutes at 60°C in water bath) was used as negative control [52].

### Immunofluorescence

The neuron-specific marker NeuN was used to confirm the identity of the cells in culture. After preparation of neuronal primary cultures, cells were fixed for 10 minutes with 2% PFA, washed with PBS, permeabilized by incubation in 0.5% Triton X-100 PBS solution and then blocked in 2% bovine serum albumin for 30 minutes. NeuN labeling was performed by overnight incubation (at 4°C) with the mouse anti-NeuN antibody (Millipore, #MAB377) diluted 1:50 in blocking solution. Following washes and incubation with the AlexaFluor 456-conjugated goat anti-mouse secondary antibody (1 hour at room temperature, 1:200), the immunoreaction was examined using an inverted Nikon Eclipse Ti confocal microscope coupled to an A1 scanning.

### Cell death assay

Neuronal cell death was determined by LIVE/DEAD Cell Viability Assay (Invitrogen, #L3224) at 6 and 24 hours upon ZIKV infection, as previously described [52,53]. Briefly, cultured neurons were stained with 2 μM of calcein acetoxymethyl ester (AM) and 2 μM of ethidium homodimer-1 for 15 minutes. Calcein-AM is permeable to the cell membrane, however, after being cleaved by esterases contained in living cells it becomes unable to cross the membrane, remaining trapped within the cell and leading to an increase of green fluorescence throughout the cell body. The ethidium-1 homodimer is only capable of permeating cells with damaged membrane, which is a consequence of the dying process. After permeating the cell membrane, the ethidium-1 homodimer binds to nucleic acids, emitting red fluorescence. Live (calcein AM^+^ cells, green staning) and dead (ethidium homodimer-1^+^ cells, red staining) neurons were imaged in the FLoid Cell Imaging Station (Thermo Scientific). A minimum of 150 cells was analyzed per well in triplicate, using ImageJ software.

### Mmu-miR-7013-5p transfection

Neuro-2A cells were plated in triplicate at 2 x 10^5^ cells/ml in 6-well plates 12 hours prior to transfection. They were transfected using 60 pmol of miRVana miRNA mimic mmu-miR-7013-5p or miRVana miRNA Mimic Negative control (miR-NC) (Thermo Fisher Scientific) and 9 μl of Lipofectamine RNAiMAX (Thermo Fisher Scientific) diluted in Opti-MEM medium (Thermo Fisher Scientific). Forty-eight hours after transfection, cells were washed twice with phosphate-buffered saline (PBS) and harvested for total protein and RNA.

### Protein extraction and Western blotting

Proteins were extracted from cells (cultured primary neuron and Neuro-2A cells) and adult mouse brain (positive control) using cold RIPA buffer [50 mM Tris pH 8.0, 150mM NaCl, 1% Nonidet (N) P-40, 5 mM EDTA pH 8.0, 0.1% sodium deoxycholate (DOC), 0.5% SDS] supplemented with phosphatase and protease inhibitor cocktail (Roche). After 20 minutes at 4°C, lysates were incubated for 30 minutes at room temperature with Benzonase-nuclease (Sigma-Aldrich) to degrade DNA and RNA. The lysates were centrifuged for 15 minutes at 12,000 g and the supernatants were collected for Western blotting.

After protein dosage by the Bradford assay (Bio-Rad), 80 μg of protein mixed with Laemmli sample buffer was incubated for 5 minutes at 95°C. Proteins were separated by sodium dodecyl sulfate polyacrylamide gel electrophoresis (SDS-PAGE) and transferred to Hybond nitrocellulose membranes using Trans-Blot turbo transfer system (BioRad). Membranes were blocked for 1 hour with 5% low-fat milk in 0.1% Tris-buffered saline tween 20 (TBS-T) followed by three washes with TBS-T [54]. The membranes were incubated overnight at 4°C with primary antibodies against NeuN (Millipore, #MAB377, 1:500), Gfap (Sigma-Aldrich, #G9269, 1:1000), Iba1 (Invitrogen, #PA5-21274, 1:1000), Npas4 (Activity Signaling, #AS-AB18A-100, 1:300), and Nr4a3 (Invitrogen, #PA5-68825, 1:1000), or for 1 hour at room temperature with an anti-β-actin antibody (Sigma-Aldrich, #A5316, 1:10000). After three washes with TBS-T, membranes were incubated with horseradish peroxidase-conjugated secondary antibodies. The immunoreaction was visualized with Western Lightning Plus-ECL reagent (Perkin-Elmer).

### RNA extraction

RNA was extracted from mock-infected and ZIKV-infected (HS-2015-BA-01) primary neurons, at 6 and 24 hours post infection (hpi) in triplicates. RNA isolated from transfected Neuro-2A cells at 48 hours was obtained in triplicates as well. The RNA extraction was carried out using TRIzol reagent (Invitrogen) and precipitated with anhydrous ethanol as described [50,55]. Total RNA resuspended in Ultrapure DNase/RNase-free distilled water (Invitrogen) was purified through miRNeasy mini kit columns (QIAGEN). RNase-free DNase set (QIAGEN) was added onto the columns to eliminate any trace of DNA. Total RNA was collected from the column using 50 μl of Ultrapure water and sent to Genome Québec, Montréal, Canada (https://cesgq.com/home) for microarray analysis. The RNA concentration was measured with a spectrophotometer/fluorometer (DeNovix, DS-11 FX+).

### Affymetrix microarray chips, Expression Console (EC) and Transcription Analysis Console (TAC) analysis

RNA derived from three different pooled samples at 3 μg/25 μl was analyzed by Genome Québec. Mouse Gene ST 2.0 arrays from Affymetrix were used for mRNA and GeneChip miRNA 4.0 (Affymetrix) Array (Mouse) for miRNAs. Microarray readouts were downloaded from Genome Québec server and analyzed with Expression Console (EC) software (Affymetrix) to assess quality metrics such as absolute deviation residuals, relative log expression and pos_neg_AUC to estimate the correct positive rate. After a quality assessment, samples were analyzed with the Transcriptome Analysis Console (TAC 3.0, Affymetrix). S1 Data shows all genes and miRNAs analyzed in Mouse Gene ST 2.0 and GeneChip miRNA 4.0 at 6 and 24 hpi.

### Reverse Transcription Quantitative Polymerase Chain Reaction (RT-qPCR)

After RNA extraction from fetal murine cells infected or mock-infected with HS-2015-BA-01 at 24 hpi, 1000 ng was utilized to synthesize complementary DNA (cDNA) using Superscript II reverse transcriptase according to the manufacturer’s protocol (Invitrogen). qPCR was carried out by diluting cDNA from mock or infected cells (1:60) after comparison with the quantification cycle (Cq) values from the standard curve of Npas4 and Nr4a genes. BrightGreen 2X qPCR MasterMix (ABM) and CFX96 thermocycler (Bio-Rad) were used to perform the qPCR assays [50,56,57]. Npas4, Nr4a1, Nr4a2 and Nr4a3 primers were designed for this work as followed:

Npas4: Forward (F) 5’ACCTAGCCCTACTGGACGTT 3’, Reverse (R) 5’ CGGGGTGTAGCAGTCCATAC3’ (99 bp product length).

Nr4a1: (F) 5’CGGCCCATTAGATGAGACCC3’, (R) 5’ GTTGGGTGTAGATGGCGAGG3’ (85 bp product length).

Nr4a2: (F) 5’TGGTTCGCACGGACAGTTTA3’, (R) 5’GGGCACTGATCAGACTCACC3’ (108 bp product length).

Nr4a3: (F) 5’AGGGCTTCTTCAAGAGAACGG3’, (R) 5’ TACTGACATCGGTTTCGGCG3’ (100 bp product length).

Primers for TATA-box binding protein (TBP) and eukaryotic translation elongation factor 2 (eEF2) were used as internal controls as previously described [57–59]. qPCR conditions were as follows: 95°C for 5 minutes followed by 49 cycles of 95°C for 10 seconds, 60°C for 15 seconds, 72°C for 5 seconds. Finally, one cycle of 65°C for 5 seconds and one of 95°C for 50 seconds. Data and statistical analysis of five replicates (n = 5) was performed using Bio-Rad CFX maestro (Bio-Rad) and GraphPad Prism 6 (GraphPad Software).

### Gene set enrichment analysis (GSEA)

Affymetrix Mouse Gene ST 2.0 arrays differential expression files from TAC 3.0 were used as input for GSEA analysis. GSEA analysis was performed using R packages ReactomePA [60] and clusterProfiler [61]. A minimum network size of 50 and a maximum of 120 were considered.

Data visualization was performed using ggplot2 [62] and pheatmap [63].

### Targetome analysis

MiRNAs with the lowest FDR at 6 and 24 hpi were analyzed with three different algorithms in different databases of predicted targets to correlate dysregulated genes of infected ZIKV neurons found in Mouse Gene ST 2.0. miRDB [64], Target scan [40,65–68] and Affymetrix TAC 3.0 were used to generate targets at 6 and 24 hpi and to identify identical target genes displayed in the gene array files (S2 Data).

### Statistical analysis

Statistical analyses for LIVE/DEAD, plaque assays and Western blots were performed in GraphPad Prism Software version 6.0 (GraphPad Software, La Jolla, CA). First, normality was assessed by Shapiro-Wilk test. Comparisons between two or more groups were performed using unpaired *t*-test or one-way ANOVA plus Tukey post hoc test, respectively. The significance level adopted for all tests was p <0.05. Except when otherwise specified, data were represented graphically as mean ± SEM.

TAC 3.0 software was used for statistical analyses of the Affymetrix microarray chips to calculate the following parameters: fold change (FC), ANOVA-p value and false discovery rate (FDR). For practical purposes, some values are presented as Log2FC. FDR ≤0.05 was used as cut-off criteria to avoid false-positive results in differentially expressed genes in mock and infected samples except where otherwise mentioned. All the aforementioned data can be consulted in S1 Data.

## Results

### ZIKV HS-2015-BA-01 infection induces cell death of fetal murine neurons at 24 hours

**Fig 1:**
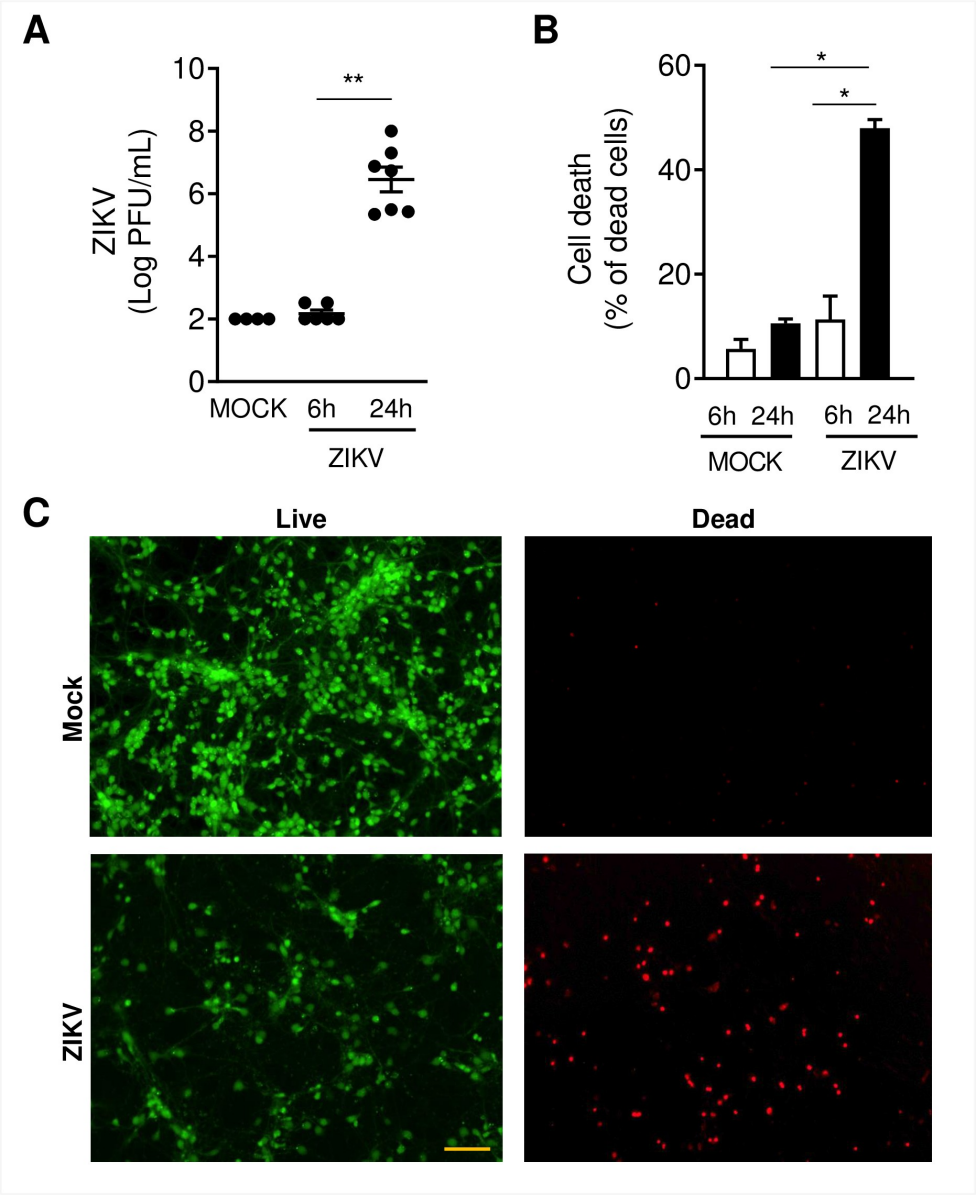
ZIKV infects primary cultured neurons and induces neuronal cell death. Primary culture from cortical-striatal C56BL/6 embryos brains (E15) on day 5 of differentiation *in vitro* (DIV5) were infected with ZIKV HS-2015-BA-01 at MOI of 1. A) Viral loads recovered from culture supernatant after 6 and 24 hours of infection with ZIKV. B) Neuronal death was assessed using the live/dead assay in primary neurons on DIV5. The graph represents the percentage of dead neurons after 6 and 24 hours of infection. C) Representative pictures from primary cultured neurons after 24 hours of ZIKV infection labeled with Calcein AM (green-live cells) and ethidium homodimer (red–dead cells). Size bar corresponds to 100 μm in all images. Results were expressed as median (A) or mean ± SEM (B). Statistically significant differences between groups were assessed in A and B by Kruskal-Wallis plus Dunn post hoc test. (*) and (**) represent a p-value < 0.05 and < 0.01, respectively.

In previous work, we have shown that primary neuronal cultures established from the cerebral cortex and striatal region of mouse brain embryos are highly permissive to ZIKV infection using the Brazilian isolate HS-2015-BA-01 (52). After confirming the purity of our cell culture system based on the expression pattern of neuronal (NeuN) and glial (Gfap and Iba1) cell markers, (S1 Fig), we infected fetal neurons with ZIKV HS-2015-BA-01 at MOI of 1. Following 6 and 24 hpi, plaque and LIVE/DEAD assays were employed to evaluate viable viral loads and cell viability, respectively. We recovered ZIKV particles from cell culture supernatants of mock- and ZIKV-infected neurons at 6 and 24 hours (Fig 1A). No viable virus was detected at 6 hpi. Nonetheless, at 24 hpi about 10^7^ viral particles were recovered in ZIKV-infected neurons. LIVE/DEAD assay results (Fig 1B) demonstrated that at 6 hpi there was no difference between ZIKV infected or mock-infected neurons. We observed at 24 hpi that ≥ 50% of the cells from the ZIKV infected group were dead. Images in Fig 1C are representative from mock and ZIKV-infected neurons labeled with Calcein AM (live cells stained in green) and ethidium homodimer (dead cells stained in red).

### ZIKV infection of fetal murine neurons induces downregulation of transcription factors

**Fig 2:**
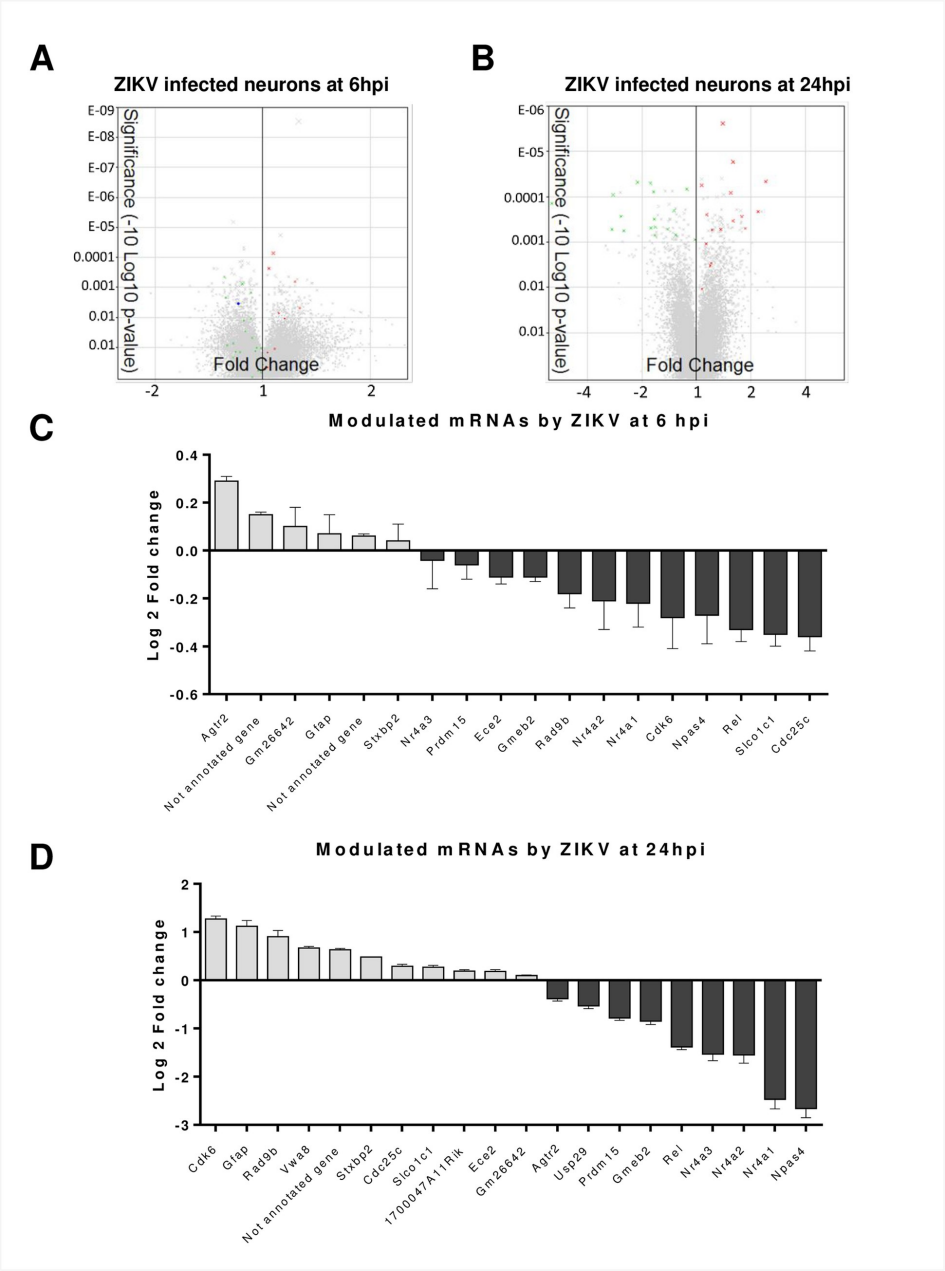
Transcriptome analysis of ZIKV infected neurons. Total RNA was collected at 6 and 24 hpi, and mRNA variations were analyzed by microarrays. A and B) Volcano plot of total differentially expressed genes after ZIKV infection at 6 hpi (A) and 24 hpi (B). Differentially expressed genes with a cut-off FDR ≤ 0.05 are in green (downregulated) and red (upregulated) dots. C) Bar plot of genes with an FDR ≤ 0.05 at 6 hpi*. Grey bars represent the upregulated genes whereas black bars are the downregulated genes. The *x*-axis shows gene names and the *y*-axis shows Log2FC. D) Bar plot of genes with an FDR ≤ 0.05 at 24 hpi*. Grey bars represent the upregulated genes whereas black bars are the downregulated genes. The *x*-axis shows gene names and the *y*-axis shows Log2FC. *Nr4a2 is shown in C and D despite an FDR value of 0.0563.

To further understand the consequences of ZIKV infection, we analyzed the transcriptome of mock-infected and infected fetal neurons. After 6 and 24 hpi at MOI of 1, we extracted RNA and performed microarrays using Affymetrix technology. We evaluated different parameters such as transcript expression, FC, ANOVA p-values, and FDR values of 34 472 genes using TAC 3.0 (S1 Data). Using an FDR ≤ 0.05 cut-off to screen reliable dysregulated genes (Fig 2A and 2B), we found distinct genes that were abnormally produced. At 6 hpi, we found 6 potential upregulated and 12 downregulated genes in comparison to mock-infected cells (Fig 2C). Because the changes in expression were not drastic, they were not considered further (S1 and S2 Data).

In contrast, most of the genes at 24 hpi with FDR values ≤ 0.05 had a more pronounced difference in transcription levels upon ZIKV infection (Fig 2D, S2 Data). Indeed, at 24 hpi we found 11 upregulated genes and 9 downregulated genes (Fig 2D). Interestingly, we found that Gm26642, Glial fibrillary acidic protein (Gfap), Syntaxin-binding protein 2 (Stxbp2), von Willebrand factor A domain-containing protein 8 (Vwa8) and 1700047A11Rik preserved their tendency to be upregulated at both times. On the other side of the graph, Ubiquitin carboxyl-terminal hydrolase 29 (Usp29), Nr4a3, PR domain zinc finger protein 15 (Prdm15), Nr4a2, Nr4a1 and Npas4 were increasingly downregulated from 6 up to 24 hpi. Nr4a3 was very weakly modulated at 6 hpi and strongly downregulated at 24 hpi. Because the Nr4a family and Npas4 are neural transcription factors [69,70] and were downregulated to the greatest extent at 24 hpi, we pursued their study and also included Nr4a2 in our analysis despite its FDR value of 0.0563 (Fig 2, S2 Data).

### Neural transcription factor Npas4 and Nr4a family are strongly downregulated by ZIKV infection

**Fig 3:**
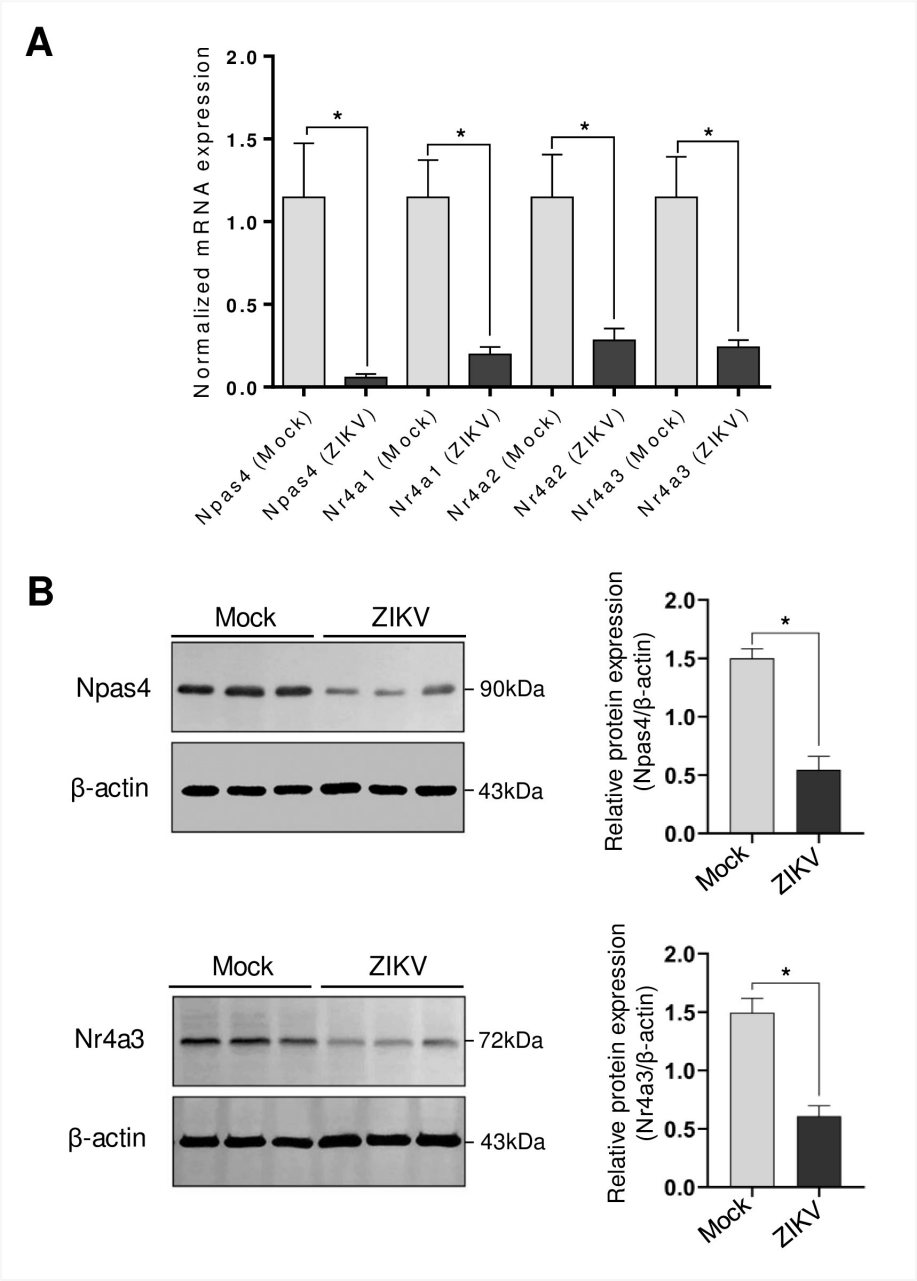
RT-qPCR and Western blotting validation for Npas4 and Nr4a family. **A)** RNA samples from uninfected and ZIKV-infected fetal murine neurons at 24 hpi were subjected to RT-qPCR using specific primers for the detection of Npas4, Nr4a1, Nr4a2 and Nr4a3 mRNAs. The graph represents mRNA expression normalized to TBP and eEF2 as internal controls and are the average of five independent experiments ± SEM. B) Western blotting assay showing reduced expression of Npas4 and Nr4a3 at 24 hpi. Figures are representative of three independent experiments. Statistically significant differences between groups were assessed in A and B by *t*-test. * represents a p-value < 0.05.

Because of the strong dysregulation of Nr4a family and Npas4 at 24 hpi, we quantified their expression by RT-qPCR and Western blots (Fig 3). Our results confirmed that Npas4, Nr4a1, Nr4a2 and Nr4a3 transcripts were profoundly decreased by ZIKV at 24 hpi. Similar to the microarray results, RT-qPCR readouts showed that Npas4 transcripts were the most affected at 24 hpi (Fig 3A). Moreover, Npas4 and Nr4a3 were also significantly downregulated at the protein level following ZIKV infection (Fig 3B). It is noteworthy that neurons infected with ZIKV-inactivated virus had levels of Npas4 and Nr4a3 comparable to mock controls (S2 Fig), thereby highlighting that downregulation of Npas4 and Nr4a3 levels is indeed dependent on virus replication. Overall, our data demonstrate that ZIKV modifies neural cellular transcription factors moderately at 6 hpi and more substantially at 24 hpi with the Nr4a family and Npas4 being the most downregulated genes.

### ZIKV infection in murine primary neurons compromises eight shared pathways at 6 and 24 hpi

**Fig 4:**
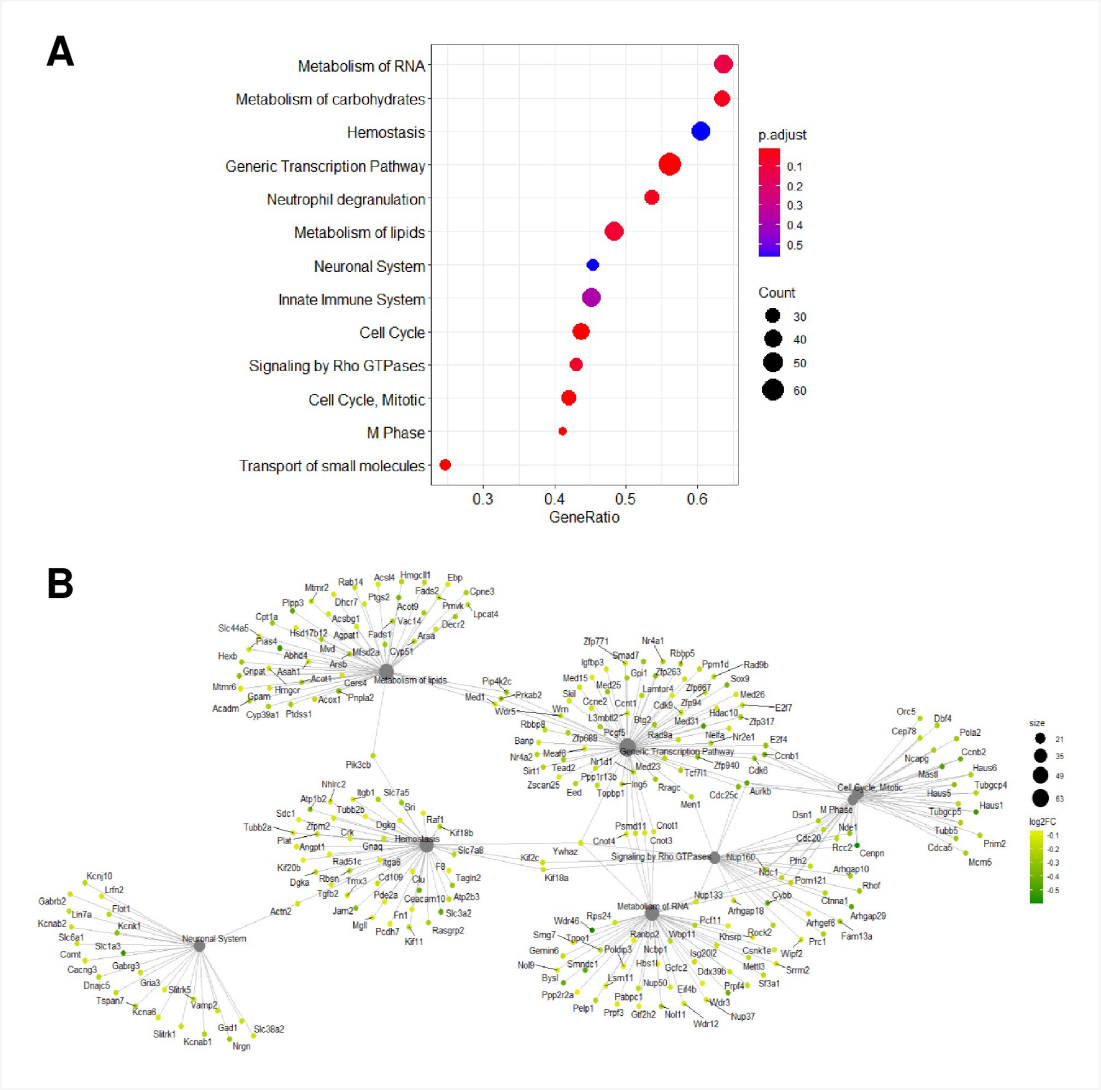
GSEA of putative mRNAs at 6 hpi with ZIKV. A) Dotplot of inferred pathways at 6 hpi. The size of the circles represents the number of dysregulated genes and their color represents the p-value. The *y*-axis displays the pathways of dysregulated genes, whereas the *x*-axis shows the fold enrichment. B) Gene-Concept Network depicting the linkages of genes and biological concepts as a network. Only core enriched genes are displayed. The color code values are on the right of the image, where yellow indicates a higher expression than green.

**Fig 5:**
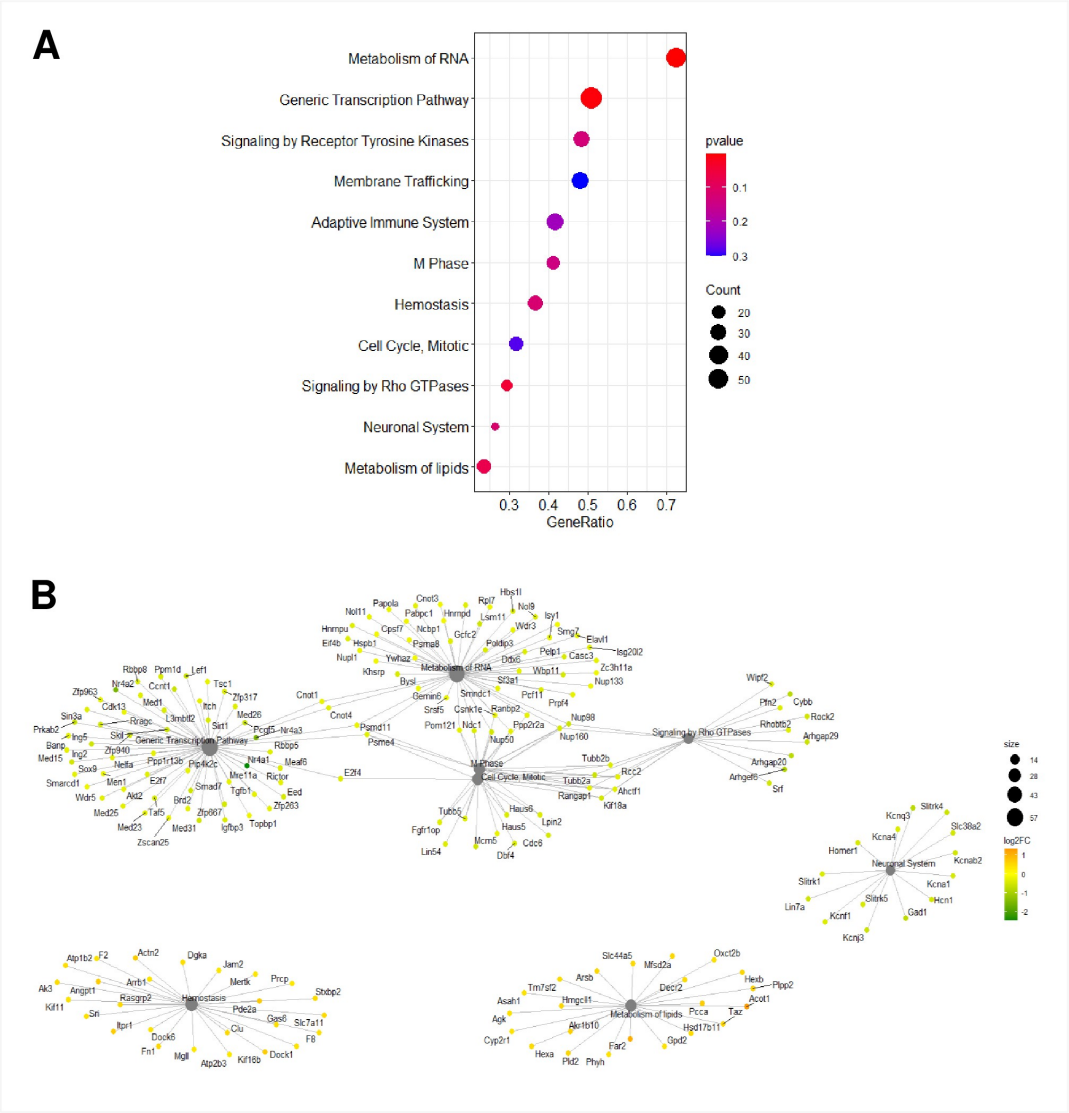
GSEA of putative mRNAs at 24 hpi with ZIKV. A) Dotplot of inferred pathways at 24 hpi. The size of the circles represents the number of dysregulated genes and their color represents the p-value. The *y*-axis displays the pathways of dysregulated genes, whereas the *x*-axis shows the fold enrichment. B) Gene-Concept Network depicting the linkage of genes and biological concepts as a network. Only core enriched genes are displayed. The color code values are on the right of the image, where orange indicates high expression and green a low one.

To investigate the correlation between the atypically regulated genes by ZIKV and the possible impact within infected cells, we performed GSEA of 34 472 genes. GSEA determines whether a set of genes is statistically altered between two biological states. We identified thirteen cellular pathways that were modified upon ZIKV infection at 6 hpi (Figs 4, S3 and S4). Furthermore, at 24 hpi, eleven cellular pathways were affected (Figs 5, S5 and S6). We identified that eight common pathways were enriched at both post-infection times: metabolism of RNA, hemostasis, generic transcription, metabolism of lipids, neuronal system, signaling by Rho GTPases, cell cycle mitotic, and M phase pathways. Further protein network analysis showed differences between dysregulated genes in common pathways at 6 and 24 hpi. Interestingly, the crossover between cellular pathways was maintained only in the generic transcription, metabolism of RNA, M phase, cell cycle, mitotic and signaling of Rho GTPases pathways (Figs 4B and 5B).

We next compared the GSEA and protein network to genes with FDR ≤ 0.05 and found that at 6 hpi downregulated genes like cell cycle checkpoint control protein RAD9B (Rad9b), Nr4a2, Nr4a1, Cyclin-dependent kinase 6 (Cdk6) and cell division cycle 25C (Cdc25c) were part of the generic transcription pathway (Figs 2C and 4B). Rad9b, and Cdk6 were also part of the cell cycle mitotic pathway and only Cd25c was included in the generic transcription, cell cycle mitotic and signaling by Rho GTPases pathways. At 24 hpi, Stxbp2 was the only upregulated gene that was within the hemostasis pathway (Fig 5B). Unlike the upregulated genes, all the Nr4a family members were pinpointed within the generic transcription pathway (Figs 2D, 3 and 5B). Our analysis suggests that ZIKV infection targets different cellular pathways, but especially the generic transcription pathway seems to be severely impaired by an important reduced expression of the Nr4a family.

### ZIKV-infected fetal murine neurons show dysregulation of specific miRNAs

**Fig 6:**
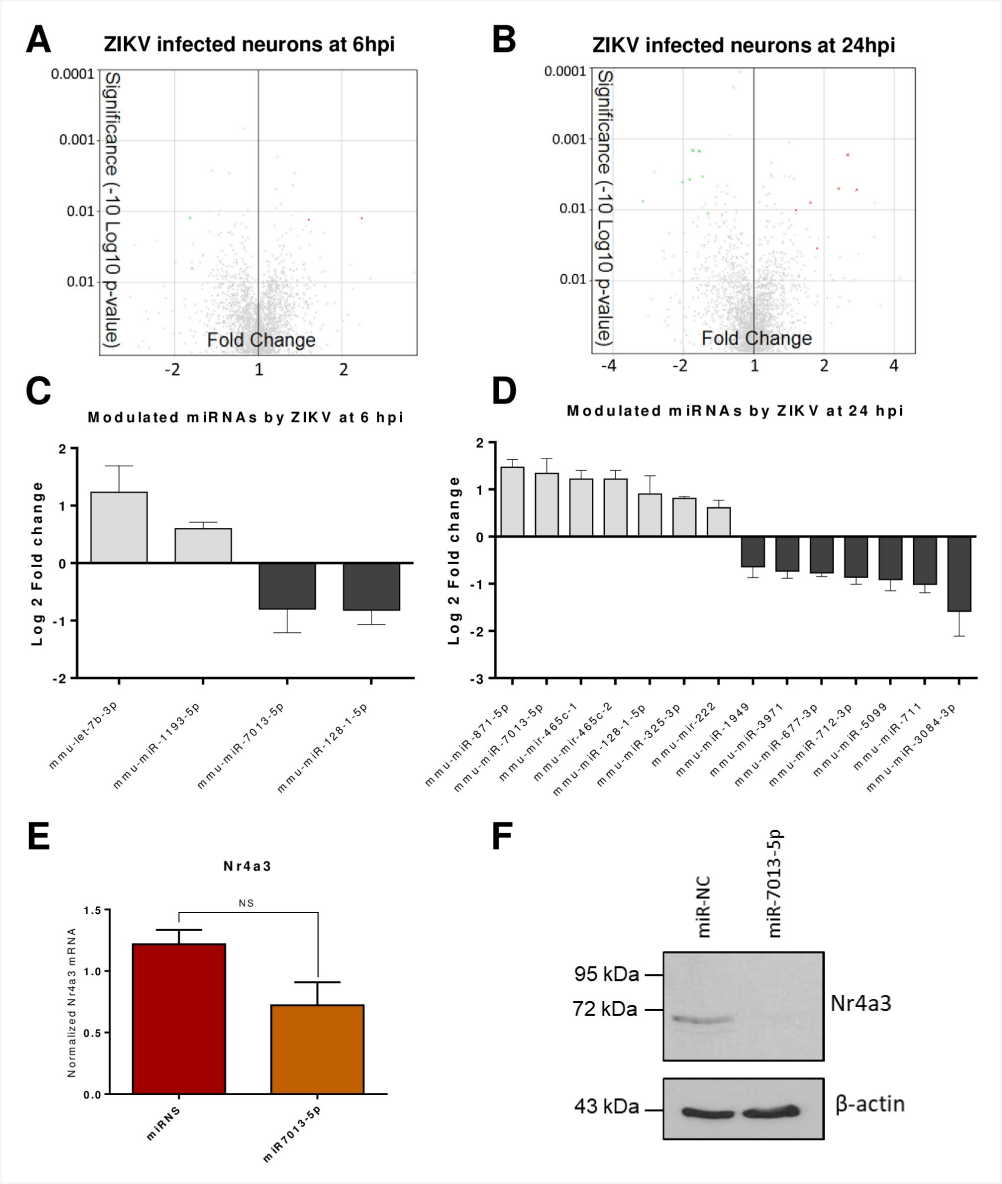
miRNA profile analysis of ZIKV infected neurons. Total RNA was collected at 6 and 24 hpi and the miRNA expression was analyzed by microarrays. A and B) Volcano plot of differentially expressed miRNAs after ZIKV infection at 6 hpi (A) and 24 hpi (B). Green (downregulated) and red (upregulated) dots represent differentially expressed miRNAs with FDR ≤ 0.5. C) Bar plot of miRNA with an FDR ≤ 0.5 at 6 hpi. Grey bars represent upregulated miRNAs whereas black bars are the downregulated miRNAs. *x*-axis shows gene names and *y*-axis shows Log2FC. D) Bar plot of miRNA with an FDR ≤ 0.5 at 24 hpi. Grey bars represent upregulated miRNAs whereas black bars are the downregulated miRNAs. *x*-axis shows gene names and *y*-axis shows Log2FC. E and F) Neuro-2A cells were transfected using 60 pmol of miR-NC as control or miR-7013-5p mimics as indicated. E) miR-7013-5p decreases Nr4a3 mRNA expression. Nr4a3 mRNA transcript levels were quantified by RT-qPCR normalized to TBP and eEF2 mRNA levels as internal controls. The graph represents the averages of mRNA Nr4a3 expression from three independent experiments. Error bars represent the SEM and *t*-test was performed to assess significance. F) miR-7013-5p decreases Nr4a3 protein expression. 80 μg of protein extracts was separated on a 10% SDS-PAGE and analyzed by Western blot using Nr4a3 antibody and anti-actin as indicated. The blot is representative of three independent experiments.

To determine whether miRNAs could contribute to the disruption of gene expression in infected neurons with ZIKV, we used Affymetrix GeneChip miRNA 4.0 arrays and TAC 3.0. We evaluated different parameters of 3195 miRNAs (S1 Data), such as transcript expression, FC, ANOVA p-values, and FDR values. The results showed modified expression of various miRNAs upon ZIKV infection, although none of them had a statistically significant dysregulation with a FDR cut-off value ≤ 0.05. Nonetheless, to identify the most dysregulated miRNAs, we selected those with a minimum of 0.5 change in Log2FC and an FDR value ≤ 0.5 (Fig 6A and 6B). At 6 hpi, we found that four miRNAs were potentially differentially expressed. MiR-Let-7b-3p and miR-1193-3p were upregulated, whereas miR-7013-5p and miR-128-1-5p had a lower expression than mock samples (Fig 6C). Several miRNA databases predicted that, miR-Let-7b-3p and miR-7013-5p possibly target Nr4a3 (Table 1, S2 Data). These results coincide with our previous findings suggesting that miR-Let7b-3p may target Nr4a2 whereas miR-7013-5p may target Nr4a3, Cdk6, Gfap, Gmeb2 and Usp29 that were dysregulated at 6h (Fig 2C).

**Table 1:** miRNA predicted dysregulated targets at 6 hpi.

*Dysregulated miRNA at 6 hpi*	*Target gene depending on each targetome database*		
	Interaction gene (TAC)	miRDB	Target scan
mmu-let-7b-3p	Nol9, Aspm, Nr4a2	Med14, Nr4a3, Nr4a2	Hist1h3d, Agtr2, Kcnq3, Med14, usp29, Nr4a3, Prdm15, Nol9, Nr4a2, Per1, Cdk6
mmu-miR-1193-5p			
mmu-miR-7013-5p		Cdk6, Gmeb2, Nr4a3	Cdk6, Gfap, Usp29, Gmeb2, Nr4a3
mmu-miR-128-1-5p			Kif16b, Stxbp2, Vwa8, Usp29, Gmeb2

We next analyzed the miRNAs affected by ZIKV at 24 hpi and detected seven potentially upregulated and seven potentially downregulated miRNAs (Fig 6D). All the predicted targets at 24 hpi are described in Table 2. Strikingly, we noticed that miR-7013-5p targets different miRNAs including Nr4a3. Because miR-7013-5p was upregulated with the lowest FDR value among miRNAs at 24 hpi and it targets Nr4a3, we pursued with further validation.

**Table 2:** miRNA predicted dysregulated targets at 24 hpi.

*Dysregulated miRNA at 24 hpi*	*Target gene depending on each targetome database*		
	Interaction gene (TAC)	miRDB	Target scan
mmu-miR-871-5p			Gfap, Vwa8, Agtr2, Csrnp1, Kcnq3
mmu-miR-7013-5p		Cdk6, Gmeb2, Nr4a3	Cdk6, Gfap, Usp29, Gmeb2, Nr4a3
mmu-mir-465c-1		Kif16b	Kif16b
mmu-mir-465c-2			Cdk6, Kif16b, Agtr2, Pak6, Csrnp1, Kcnq3, Nr4a3, Nr4a2
mmu-miR-128-1-5p			Kif16b, Vwa8, Stxbp2, Usp29, Gmeb2
mmu-miR-325-3p		Cdk6	Cdk6, Csrnp1, Kcnq3
mmu-mir-222		3p (Kif16b)	5p (Hist1h3d, Stxbp2, Gmeb2, Nr4a1). 3p Kif16b
mmu-miR-1949		Nr4a2	Kif16b, Med14, Kcnq3, Nr4a2,
mmu-miR-3971		Cdk6, Nr4a3	Cdk6, Hist1h3d, Usp29, Csrnp1, Kcnq3, Nr4a3
mmu-miR-677-3p		Usp29	Cdk6, Kif16b, Ece2, Agtr2, Usp29, Pak6, Csrnp1, Kcnq3, Prdm10, Per1
mmu-miR-712-3p			Vwa8
mmu-miR-5099			Med14, Kcnq3
mmu-miR-711	Slc27a1		Prdm15
mmu-miR-3084-3p		Cdk6, Prdm15, Nr4a2	Cdk6, Gfap, Stxbp2, Usp29, Prdm15, Kcnq3, Gmeb2, Per1, Nr4a3, Nr4a2

### MiR-7013-5p downregulates the early neuronal transcription factor Nr4a3

Considering that miR-7013-5p was highly upregulated at 24 hpi (Fig 6C and 6D) and its predicted targets Nr4a3, a neuronal immediate early gene, was highly downregulated at 24 hpi, we investigated a possible regulation of Nr4a3 by this miRNA. We overexpressed a miR-7013-5p mimic in the murine Neuro-2A cells to determine if it could downregulate Nr4a3 expression. By measuring Nr4a3 mRNA transcripts by RT-qPCR, we identified that the overexpression of miR-7013-5p reduced Nr4a3 mRNA levels by 40% (Fig 6E). Because miRNAs mostly act by inhibiting translation, we next investigated if this miR-7013-5p mimic could decrease Nr4a3 protein expression. By Western blot analysis, we observed that Nr4a3 protein expression was severally impaired after transfection of miR-7013-5p (Fig 6F). These experiments show that a ZIKV-induced miRNA can directly inhibit the expression of the neuronal gene Nr4a3.

## Discussion

Npas4 and Nr4a genes are part of the neural immediate-early genes (IEG), which can be stimulated within minutes in different regions of the brain in response to physiological stimuli (calcium influx, nerve growth factors) or pathological stimuli (inflammatory agents like TNF-α or lipopolysaccharide) [69,70]. Npas4 is a cell-specific gene, only transcribed in neurons, which regulates the response of neuronal excitation through the balance of inhibitory and excitatory synapses [71]. The disruption of this balance is related to neurological disorders such as schizophrenia, autism, anxiety and depression [72,73]. Npas4 plays a key role during neuronal development and differentiation in embryonic and postnatal growth [74–76]. Npas4 influences approximately 300 genes, amongst which more than half are linked to neurological activity [71]. Our very selective ZIKV infection of mice fetal neurons with the Brazilian strain HS-2015-BA-01 (Figs 1 and S1) induced the downregulation of Npas 4 transcription from a -0.27 Log2FC at 6 hpi to a -2.66 Log2FC at 24 hpi in microarrays (Fig 2C and 2D; S1 Data). This severe downregulation was confirmed by RT-qPCR and Western blotting in separate experiments (Fig 3).

Npas4 was not linked to any cellular pathways in our GSEA analysis, therefore we sought Npas4 and alternative names (NXF; Le-PAS; PASD10; bHLHe79) in the Reactome database but found no hits in the database itself. Npas4’s function has not been fully explored and it is likely that a definite cellular pathway does not yet exist for this gene in Reactome. Nevertheless, this gene is considered a calcium-dependent transcription factor that modulates multiple functions such as transcription, G-protein signaling, kinases and phosphatase activities, ubiquitination and endocytosis and we can anticipate that its dysregulation will induce profound modifications in brain development and functions [77].

The IEGs, Nr4as (Nr4a1, Nr4a2 and Nr4a3) like Npas4 play an essential role in the homeostasis and response due to external stimuli [69,70]. Unlike Npas4, Nr4as are expressed in different tissues and behave differently. For instance, Nr4a1 (also called Nur77) induces apoptosis in T-cells and macrophages [70,78,79]. Under certain stimuli, Nr4a1 translocates from the nucleus to the mitochondrial outer membrane where it encounters the apoptosis regulator Bcl-2. Nr4a1 associates to the N-terminal region of Bcl-2 exposing its pro-apoptotic BH3 domain, which consequently decreases the antiapoptotic activity of Bcl-xL causing downstream mechanisms that trigger apoptosis [80]. The observed decrease of Nr4a1 and its involvement in the transcription pathway (Figs 2C, 2D, 3A and 5B) may be related to a ZIKV survival mechanism to avoid the induction of apoptosis and autophagy.

Nr4a2 (Nurr1) plays an essential role as a neuroprotector, and mutations in this gene or its absence are associated with dysfunction in neuronal development and chronic pathologies like Parkinson’s disease [81,82]. Studies in T cells also indicate that Nr4a2 plays a role in homeostasis by its association with Foxp3, which is expressed in regulatory T cells (Tregs). Tregs play a role in immunological self-tolerance in autoimmune diseases and allergies [83]. The Nr4a2-Foxp3 association is related to the repression of cytokine expression including interferon (IFN)-γ. The absence or disruption of Nr4a2 is linked to atypical polarization to Th1 response and the exacerbation of inflammatory diseases [84]. These facts open the question of whether Nr4a2 decrease could be linked to the expression of IFN or other innate responses in neurons (Figs 2C, 2D, 3A and 5B).

Nr4a3 (NOR-1) has similar characteristics to the other members of the Nr4a family. It is an abundantly expressed gene in neurons and its reduction significantly affects neuronal survival and axon guidance [85]. The removal of this gene in some murine models has shown that it can be considered an essential gene in mouse embryogenesis [86]. Other knock-out mouse models have reported abnormalities during hippocampal development [85]. Exogeneous stress of chondrocytes triggers the overexpression of Nr4a3, which consequently enhances the production of pro-inflammatory interleukins like IL-1β [87]. In contrast, Tregs express high levels of Nr4a3 and similarly to Nr4a2, the protein can bind to Foxp3 and repress IFN-γ [84]. Nr4a3 is downregulated after ZIKV infection of mice fetal neurons as identified in our microarrays, a feature that was confirmed at the RNA and the protein levels (Figs 2C, 2D and 3). It is present in the transcription pathway at 24 hpi (Fig 5), which suggests a contribution to the neuron cytopathicity in concert with the other Nr4a members.

In contrast to the downregulated genes, we observed a substantial upregulation of several genes from 6 hpi to 24 hpi including Gfap, Vwa8 and Stxbp2 (Fig 2C and 2D). Notably, Gfap was the most increased gene at 24 hpi that was found at both infection times. Gfap is considered a specific marker for glia. Although Gfap expression has been documented in hippocampal neurons and neuronal progenitors where it has been linked to the rise of immature neurons [88–90], we only observed this increase in microarrays. In line, herein we did not detect Gfap protein expression by Western blotting in cultured neurons, even following 24 hours of ZIKV infection. This finding suggests that ZIKV-induced Gfap expression does not occur at the protein level and rules out a glial contamination (S1 and S7 Figs) as previously observed in neuronal cultures [52].

Multiple factors can influence the abnormal regulation of genes after viral infection. MiRNAs are master regulators of post-transcriptional gene expression leading to the control of cell physiology and the ability to respond to viral challenges [91–94]. In neurons, miRNA activity is critical in different processes like synapse development, axon guidance, neurogenesis and aging [95–98]. By analyzing the expression levels of 3195 miRNAs, we identified several changes in their expression during ZIKV infection (Figs 6 and S1 and S2 Data). We then associated the miRNAs with changes more than ±0.5 log2FC, and FDR ≤ 0.5 with filtered genes using different miRNA prediction databases (Tables 1 and 2).

Among the upregulated miRNAs, miR-7013-5p and miR-128-1-5p had a significant change, especially miR-7013-5p. Because miR-7013-5p was remarkably changed, had the lowest FDR at both times and targeted one of the most downregulated genes in our analysis (Nr4a3), we validated its predicted value in mouse neuroblasts Neuro-2A using miR-7013-5p mimics. Indeed miR-7013-5p reduced Nr4a3 expression moderately at the transcription level and more importantly at the translation level (Fig 6E and 6F) demonstrating a link between decreased gene expression and increased miRNA expression.

Few studies have performed a correlation between mRNAs and miRNAs in ZIKV-infected cells. Next-Generation sequencing (NGS) revealed an upregulation of different miRNAs with antiviral properties in an astrocytic cell line infected with a ZIKV strains from Puerto Rico. This study also pointed out to the increased expression of genes involved in the unfolded protein response pathway in the ER, but no validation was performed at the mRNA or protein level [47]. Using the same ZIKV strain to infect neurons from newborn mice and nanoString nCounter gene expression assay another group showed increased expression of genes from the antiviral immunity, inflammation and apoptosis. Some miRNAs were also modulated by ZIKV infection with a predicted role in neuroinflammation pathway, but no impact on the targeted genes was verified [48]. NGS was also performed to identify dysregulated mRNAs and miRNAs in human neuronal stem cells (NSC) infected with an African and a Brazilian strain of ZIKV. The regulatory interaction network suggested a miRNA repression of genes involved in cell cycle, stem cell maintenance and neurogenesis. Downstream analysis of Argonaute-bound RNAs identified a correlation between upregulated let-7c and downregulation of its predicted target HMGA2. Similarly, the Argonaute-bound miR-124-3p expression was increased, while its predicted target transferrin receptor (Tfrc) mRNA involved in stem cell maintenance was decreased in NSCs and in ZIKV-infected mice [49]. None of the aforementioned miRNAs and mRNAs identified in these studies was highly modified in our results, although they were present on the gene array but we have observed others. Furthermore, the most abnormal regulated genes identified in our studies were validated at the mRNA and at the protein level with a directly characterized downregulation of a gene by an upregulated miRNA. Whereas differences in cells, in ZIKV strains, techniques and bioinformatics could explain some of the discrepancies, our results indicate a profound defect of the expression of neural transcription factors in embryonic mice neurons that was not observed in other studies. Although these results would have to be corroborated in post-mortem human samples of fetuses who did not survive—which is much more difficult to obtain—they provide useful information into molecular explanations to congenital defects in fetal brain leading to CZVS. Future studies will determine the biological consequences of the changes herein portrayed.

In conclusion, our results show that ZIKV infection induces an aberrant downregulation of the neural transcription factors Npas4 and Nr4as that affect the generic transcription pathway in neurons. Our integrative analysis and validation indicate that Nr4a3 is downregulated by miR-7013-5p, which is upregulated during ZIKV infection of neurons. Overall our results link ZIKV infection of fetal neurons and neuronal gene dysregulation, which contributes to a better comprehension of neuronal pathogenesis caused by ZIKV.

## Supporting information

S1 Data Microarray raw data and differential analysis. Raw microarray data using TAC 3.0. Different parameters are displayed in the different excel sheets. From left to right: Cluster ID, average signal, fold change (FC), ANOVA-p value, False discovery rate (FDR), gene symbol and gene description. Sheets one and two represent Mouse Gene ST 2.0 array mRNA analysis at 6 and 24 hpi compared to mock respectively. Sheets 3 and 4 represent GeneChip miRNA 4.0 array miRNA analysis at 6 and 24 hpi compared to mock respectively.
(XLSX)

S2 Data FDR filtered differentially expressed mRNA and miRNA. Sheets one and two represent Log2FC of mRNA targets with FDRs less than 0.05 (and Nr4a2) at 6 and 24 hpi compared to mock respectively. Sheets three and four represent Log2FC of miRNA targets with FDRs less than 0.5 at 6 and 24 hpi compared to mock respectively along with predicted target genes using TAC, miRDB and target scan. The predicted targets are also shown in Tables 1 and 2.
(XLSX)

S1 Fig Primary cultures from C56BL/6 brain embryos are composed predominantly by neurons. Fetal neuronal cultures were prepared from the cerebral cortex and striatal regions of mouse embryos (E15) and checked for the expression of neuronal and glial cell markers. A) Immunofluorescence assay showing that cultured cells stain for the neuron-specific marker NeuN. Lower insert represents negative control. B) Western blotting highlighting the absence of expression of astrocyte (Gfap) and microglia (Iba1) markers in the cultured neuronal system. Brain protein extract was used as positive control. The pictures are representative of three independent assays.
(TIF)

S2 Fig Western blotting assays showing comparable levels of Npas4 and Nr4a3 between Mock and ZIKV-inactivated controls. Neuronal cultures were inoculated with ZIKV-inactivated virus (at 60°C for 30 minutes) or control medium (Mock). Supernatant and cell lysates were collected after 24 hours for plaque assay (A) and assessment of Npas4 and Nr4a3 protein levels (B), respectively. Note that both control groups display similar expressions of Npas4 and Nr4a3. The pictures are representative of two independent assays.
(TIF)

S3 Fig GSEA of putative mRNAs at 6 hpi with ZIKV. Enrichment score plots of ranked genes at 6 hpi with ZIKV HS-2015-BA-01. The name of each cellular pathway is on top. The *x*-axis shows the rank in order in each dataset. *y*-axis: the top panel displays the enrichment score and contains a green line denoting the enrichment score whereas the black bars represent the leading edge subset. The bottom panel shows the value of the ranking metric, which measures the gene’s correlation with a phenotype.
(TIF)

S4 Fig Gene-Concept Network at 6 hpi with ZIKV. Gene-concept depicting the linkages of genes and biological concepts as a network. All core enriched genes at 6 hpi are displayed. The color code values are on the right of the image, where yellow indicates more expression and green less expression.
(TIF)

S5 Fig GSEA of putative mRNAs at 24 hpi with ZIKV. Enrichment score plots of ranked genes at 24 hpi with ZIKV HS-2015-BA-01. The name of each cellular pathway is on top. The *x*—axis shows the rank in order in each dataset. *y*-axis: the top panel displays the enrichment score and contains a green line denoting the enrichment score whereas the black bars represent the leading edge subset. The bottom panel shows the value of the ranking metric, which measures the gene’s correlation with a phenotype.
(TIF)

S6 Fig Gene-Concept Network at 24 hpi with ZIKV. Gene-concept depicting the linkages of genes and biological concepts as a network. All core enriched genes at 24 hpi are displayed. The color code values are on the right of the image, where orange indicates more expression and green less expression.
(TIF)

S7 Fig Evaluation of Gfap expression in neuronal cultures infected with ZIKV. Fetal neuronal cultures were infected with ZIKV and the cell lysates were processed after 24 hours for Western blotting. Besides Mock, neuronal cultures infected with inactivated ZIKV virus as well as protein extract from adult mouse brain were used as control samples. The immunoblot is representative of three independent assays.
(TIF)
