# Supplementary figures and images for: Profound downregulation of neural transcription factor Npas4 and Nr4a family in fetal mice neurons infected with Zika virus
Source: PLoS Negl Trop Dis. 2021 May 28;15(5):e0009425. doi: 10.1371/journal.pntd.0009425 (PMC8191876; doi:10.1371/journal.pntd.0009425)

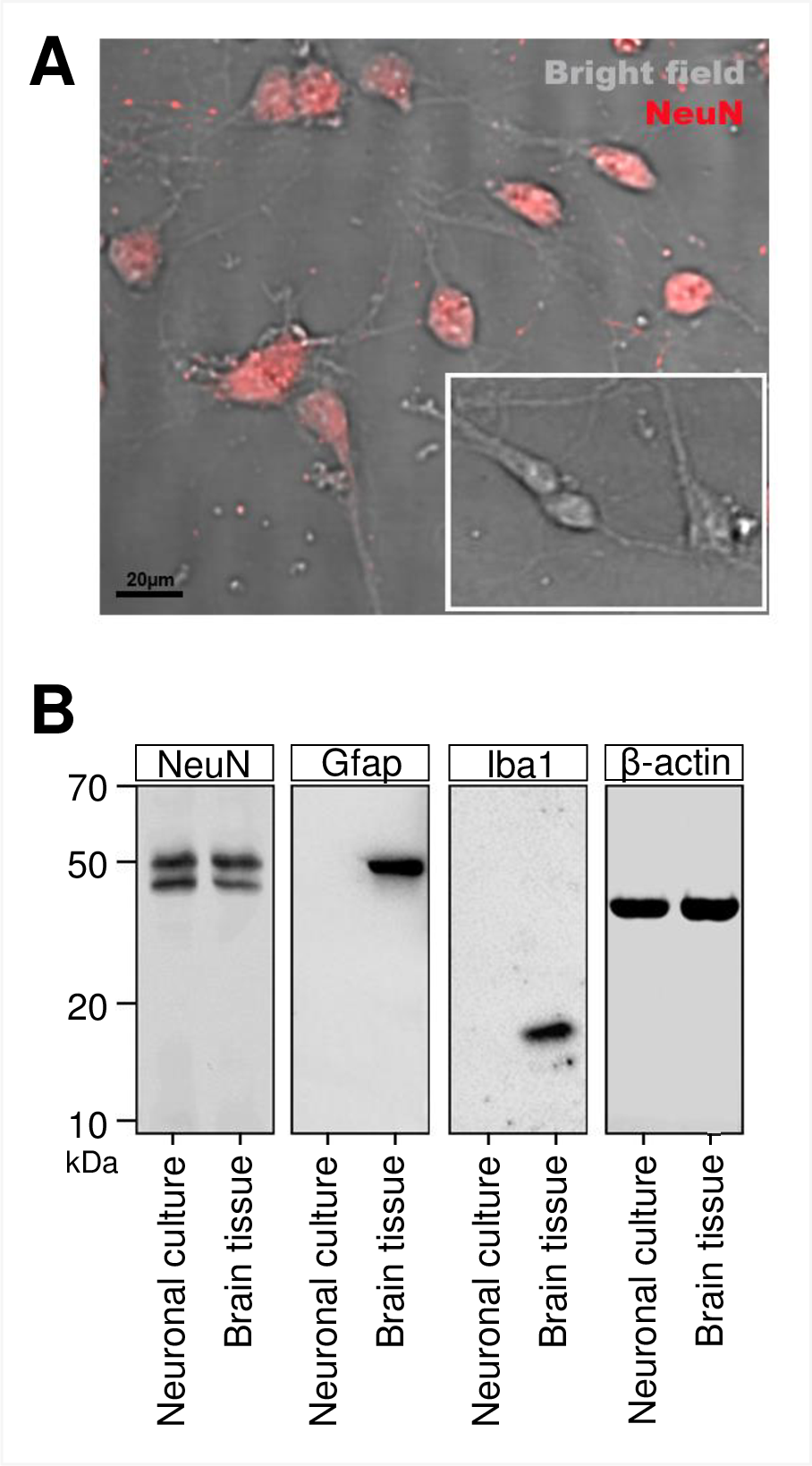

Supplement: S1 Fig — Fetal neuronal cultures were prepared from the cerebral cortex and striatal regions of mouse embryos (E15) and checked for the expression of neuronal and glial cell markers. A) Immunofluorescence assay showing that cultured cells stain for the neuron-specific marker NeuN. Lower insert represents negative control. B) Western blotting highlighting the absence of expression of astrocyte (Gfap) and microglia (Iba1) markers in the cultured neuronal system. Brain protein extract was used as positive control. The pictures are representative of three independent assays. (TIF) [file pntd.0009425.s003.tif]

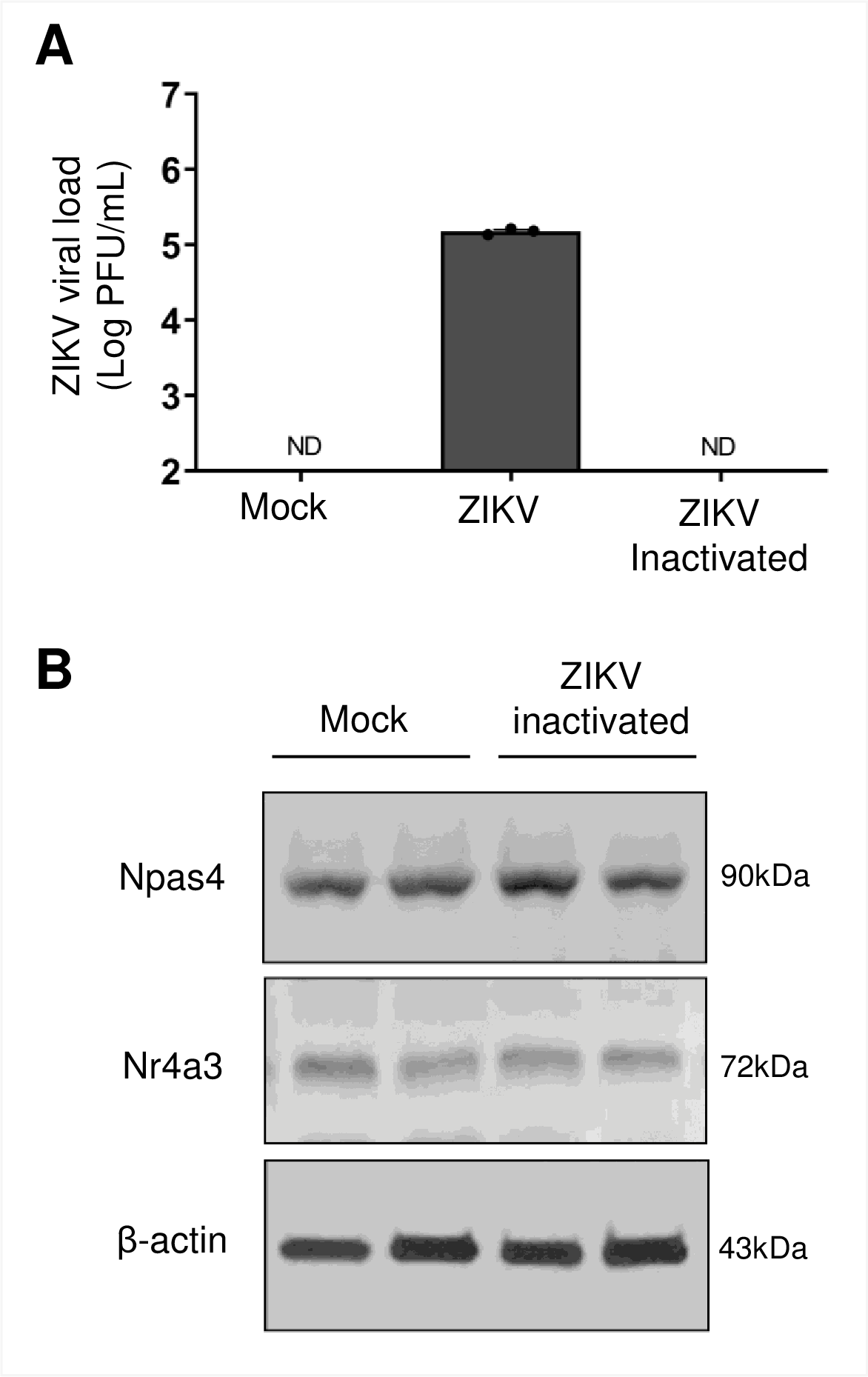

Supplement: S2 Fig — Neuronal cultures were inoculated with ZIKV-inactivated virus (at 60°C for 30 minutes) or control medium (Mock). Supernatant and cell lysates were collected after 24 hours for plaque assay (A) and assessment of Npas4 and Nr4a3 protein levels (B), respectively. Note that both control groups display similar expressions of Npas4 and Nr4a3. The pictures are representative of two independent assays. (TIF) [file pntd.0009425.s004.tif]

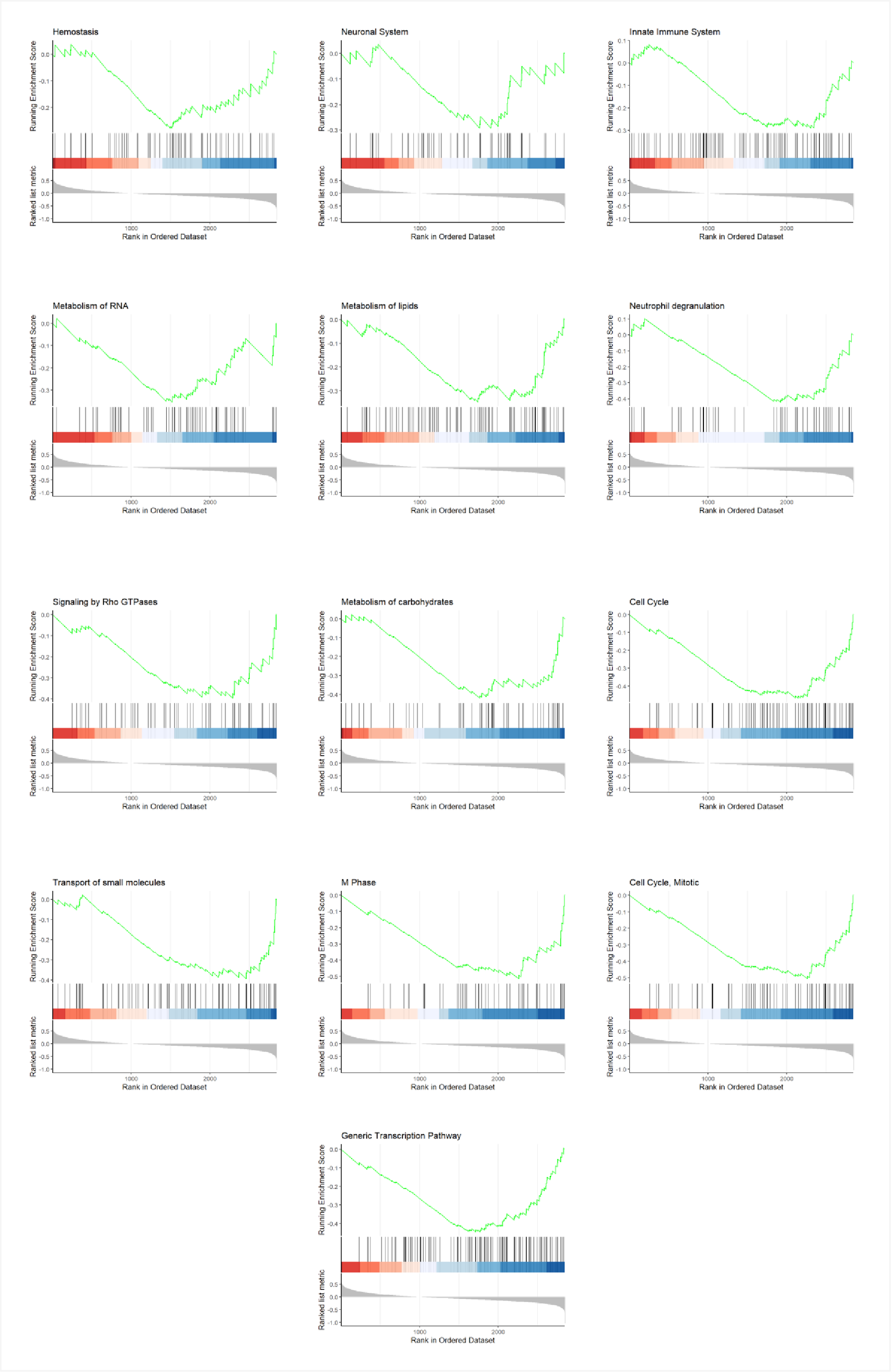

Supplement: S3 Fig — Enrichment score plots of ranked genes at 6 hpi with ZIKV HS-2015-BA-01. The name of each cellular pathway is on top. The x-axis shows the rank in order in each dataset. y-axis: the top panel displays the enrichment score and contains a green line denoting the enrichment score whereas the black bars represent the leading edge subset. The bottom panel shows the value of the ranking metric, which measures the gene’s correlation with a phenotype. (TIF) [file pntd.0009425.s005.tif]

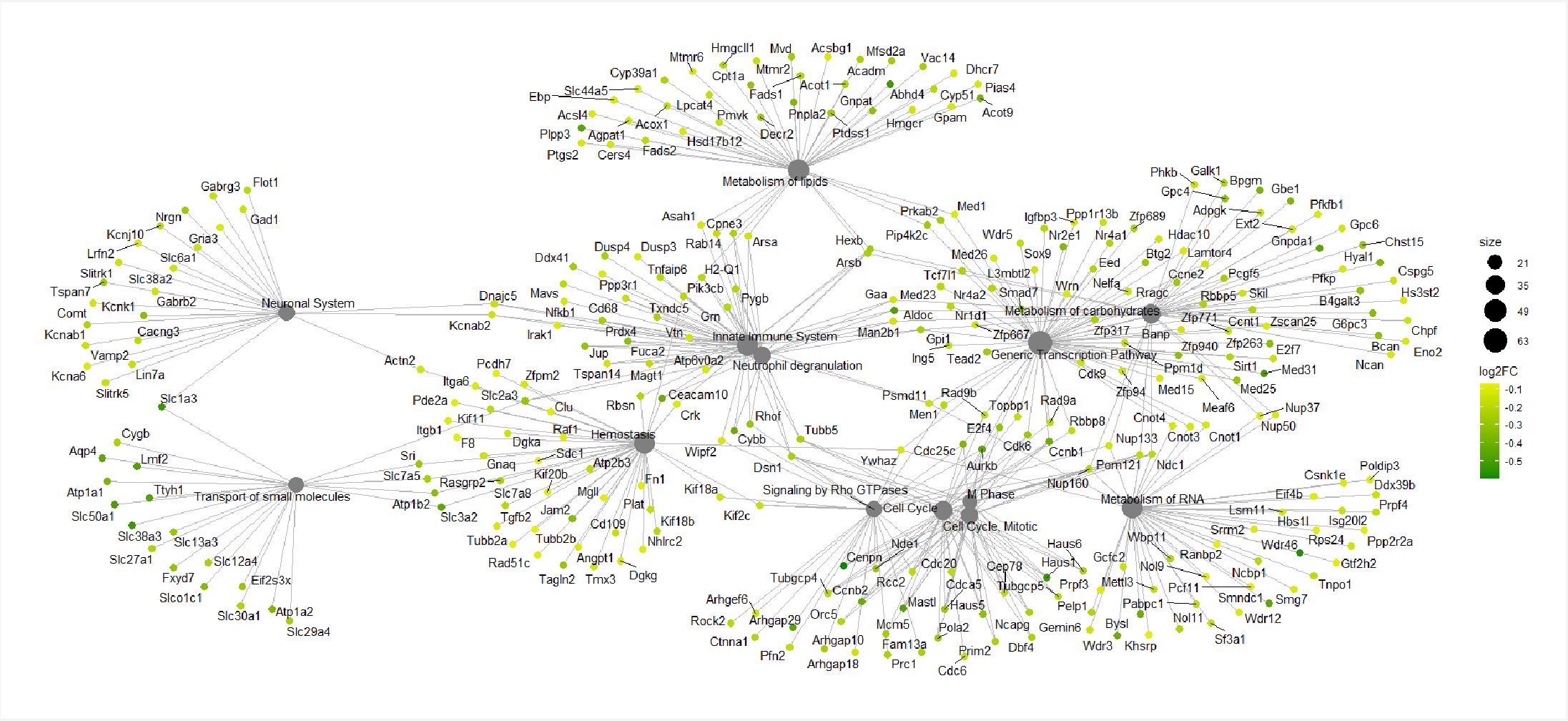

Supplement: S4 Fig — Gene-concept depicting the linkages of genes and biological concepts as a network. All core enriched genes at 6 hpi are displayed. The color code values are on the right of the image, where yellow indicates more expression and green less expression. (TIF) [file pntd.0009425.s006.tif]

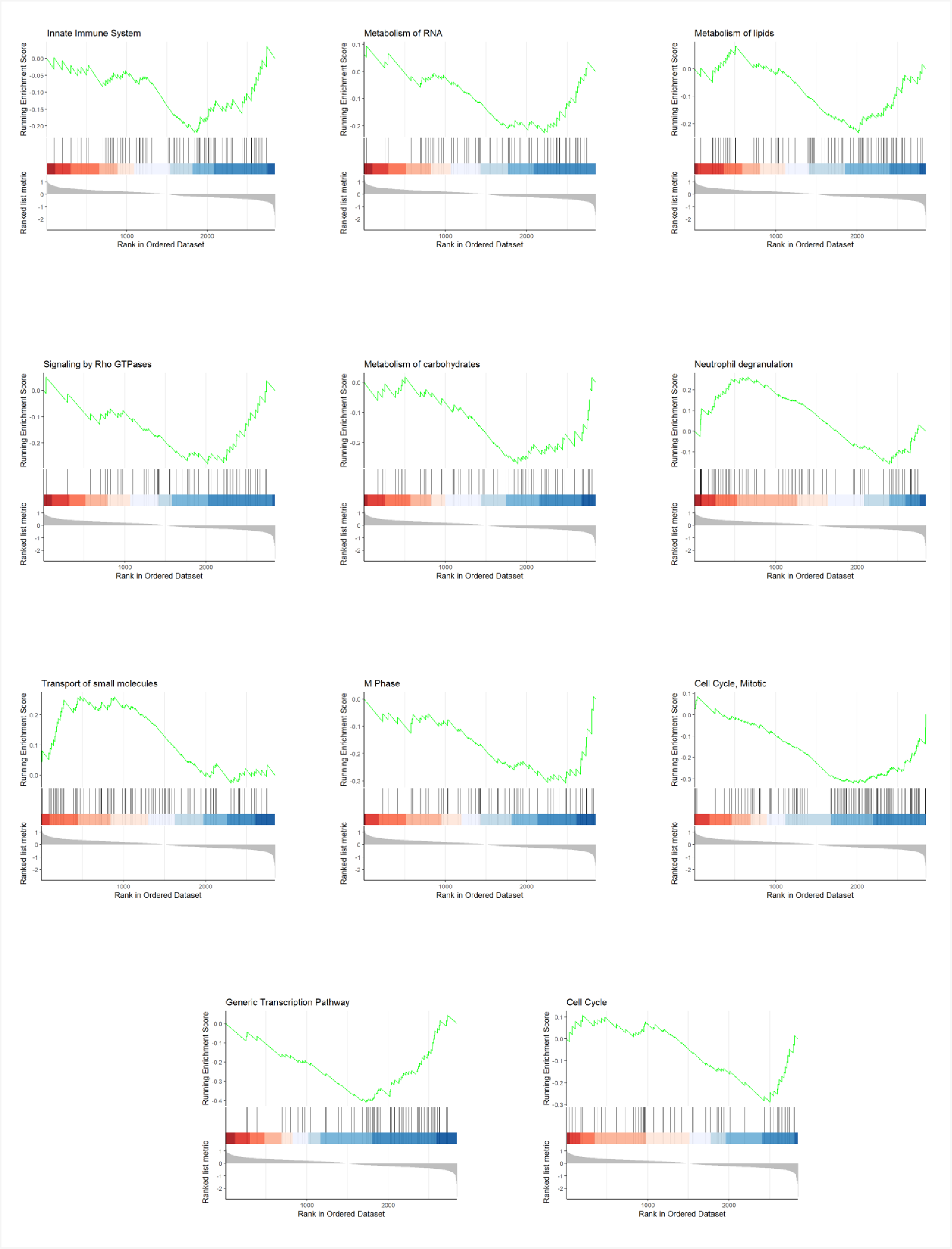

Supplement: S5 Fig — Enrichment score plots of ranked genes at 24 hpi with ZIKV HS-2015-BA-01. The name of each cellular pathway is on top. The x—axis shows the rank in order in each dataset. y-axis: the top panel displays the enrichment score and contains a green line denoting the enrichment score whereas the black bars represent the leading edge subset. The bottom panel shows the value of the ranking metric, which measures the gene’s correlation with a phenotype. (TIF) [file pntd.0009425.s007.tif]

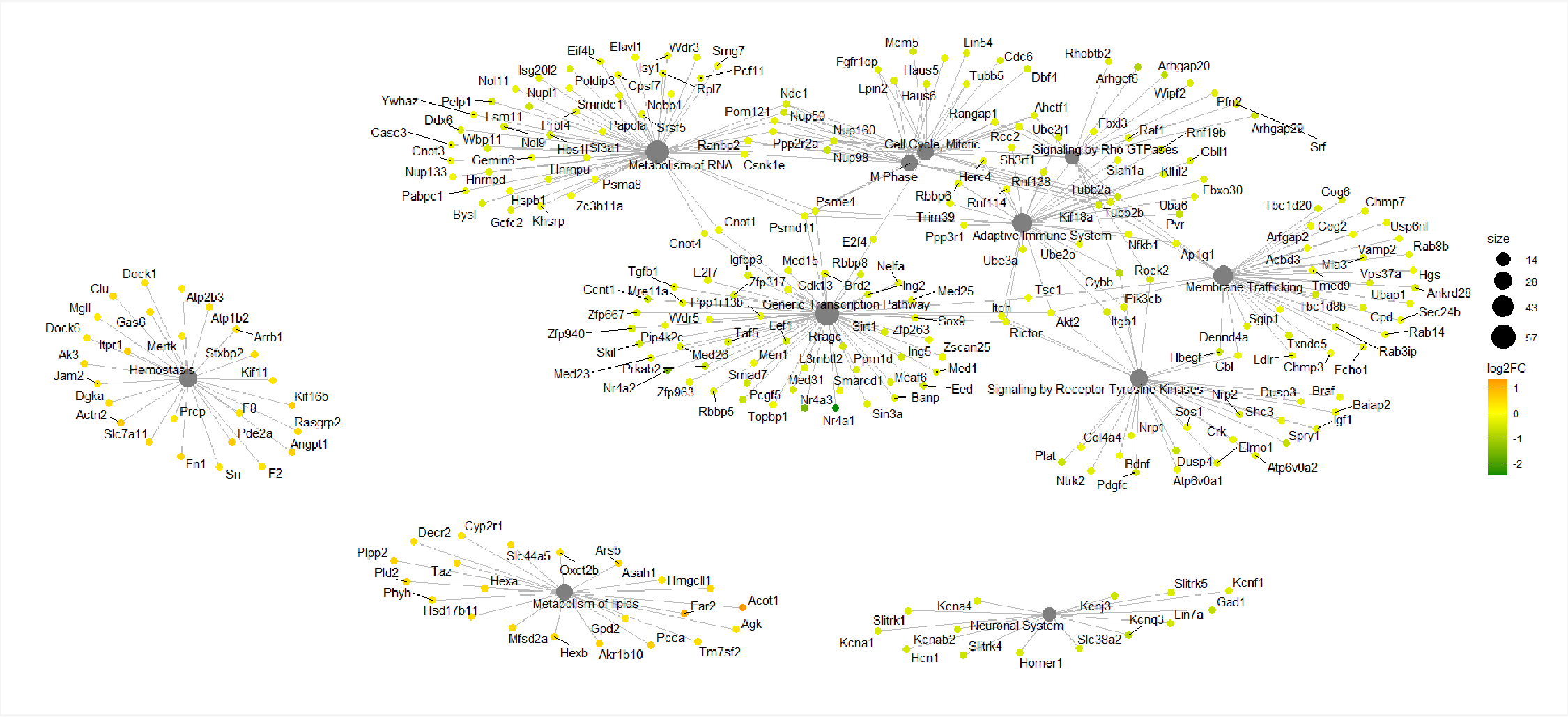

Supplement: S6 Fig — Gene-concept depicting the linkages of genes and biological concepts as a network. All core enriched genes at 24 hpi are displayed. The color code values are on the right of the image, where orange indicates more expression and green less expression. (TIF) [file pntd.0009425.s008.tif]

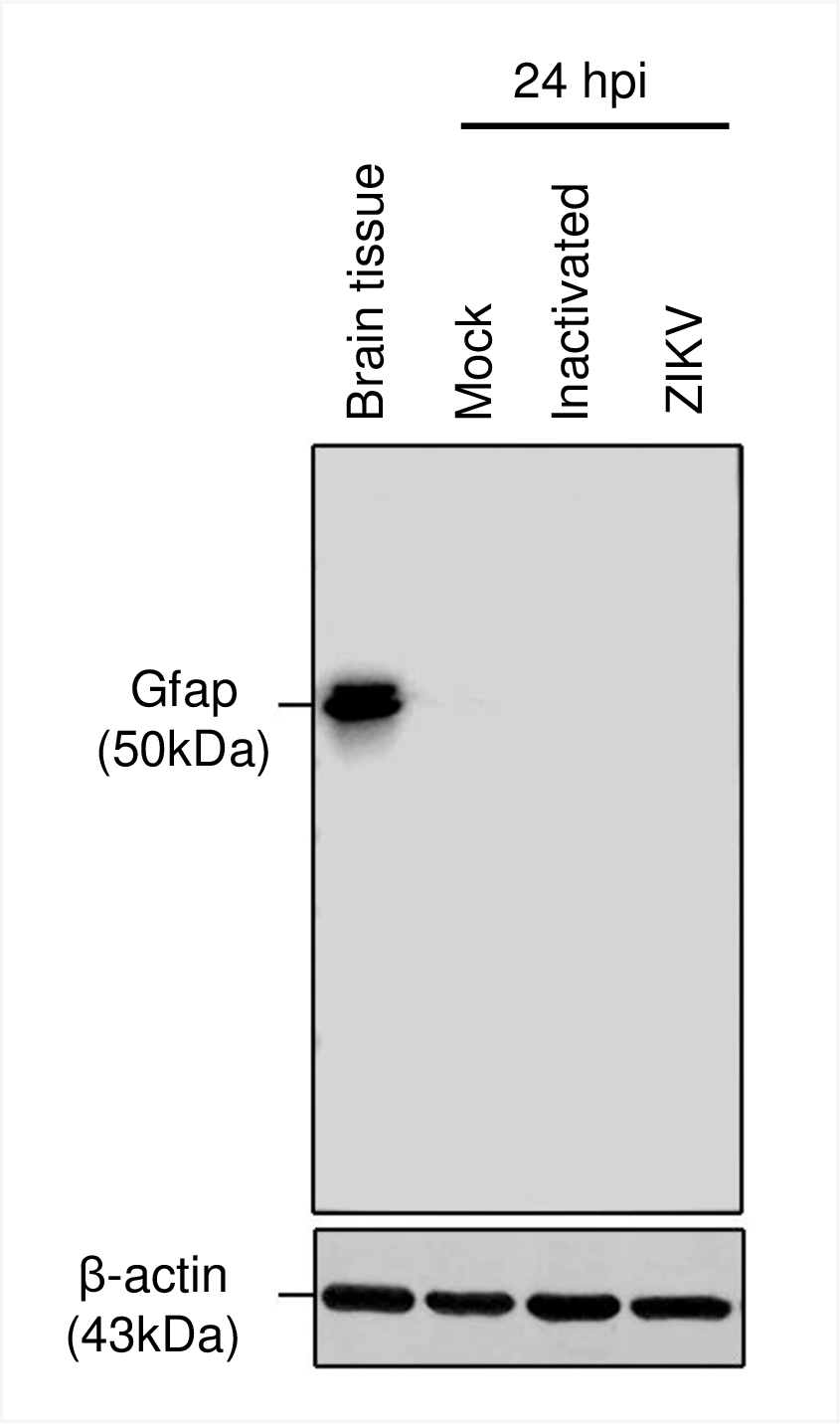

Supplement: S7 Fig — Fetal neuronal cultures were infected with ZIKV and the cell lysates were processed after 24 hours for Western blotting. Besides Mock, neuronal cultures infected with inactivated ZIKV virus as well as protein extract from adult mouse brain were used as control samples. The immunoblot is representative of three independent assays. (TIF) [file pntd.0009425.s009.tif]
